# Commentary: Early-in-life Isoflurane Exposure Alters Resting-State Functional Connectivity in Juvenile Non-human Primates - a Role for Neuroinflammation?

**DOI:** 10.29245/2578-3009/2024/2.1255

**Published:** 2024-05-17

**Authors:** Viola Neudecker, Jose F. Perez-Zoghbi, Ansgar M. Brambrink

**Affiliations:** 1 Department of Anesthesiology, Columbia University Medical Center, New York, NY, USA

**Keywords:** Neuroinflammation, astrogliosis, neurodevelopment, non-human primates, brain functional connectivity, anesthesia-induced developmental neurotoxicity

## Abstract

The concern about anesthesia-induced developmental neurotoxicity (AIDN) in infants and young children arises from animal studies indicating potential long-term neurobehavioral impairments following early-in-life anesthesia exposure. While initial clinical studies provided ambiguous results, recent prospective assessments in children indicate associations between early-in-life anesthesia exposure and later behavioral alterations. Ethical constraints and confounding factors in clinical studies pose challenges in establishing a direct causal link and in investigating its mechanisms. This commentary on a recent study in non-human primates (NHPs) focuses on exploring the role of neuroinflammation and alterations in brain functional connectivity in the behavioral impairments following early-in-life anesthesia exposure. In juvenile NHPs, chronic astrogliosis in the amygdala correlates with alterations in functional connectivity between this area with other regions of the brain and with the behavioral impairments, suggesting a potential mechanism for AIDN. Despite acknowledging the study’s limitations, these findings emphasize the need for further research with larger cohorts to confirm these associations and to establish a causal link between the neuroinflammation and the behavioral alterations associated with early-in-life anesthesia exposure.

## Anesthesia-induced developmental neurotoxicity (AIDN)

There is a concern that anesthesia exposure early in life may be neurotoxic to infants and young children and cause long-term impairments in their neurobehavioral development. This concern originated from preclinical studies in rodents, which found that early-in-life exposure to anesthetics commonly used in pediatric anesthesia caused acute increase of neuroapoptosis and long-term alterations in cognition and behaviors^1^. Most of these findings also have been observed in non-human primates (NHPs)^2–8^. In contrast, early clinical studies with retrospective assessments provided ambiguous results, with some suggesting that anesthesia exposure affected primarily cognitive abilities,^9–13^ while others found no associations. However, most recent clinical studies with prospective assessments have provided some valuable outcomes that indicate an association between early-in-life anesthesia exposure and alterations in behavior later in life,^14–16^ which have been highlighted in a recent meta-analysis^17^. Ethical limitations and the presence of numerous confounders in clinical studies make it a challenge to establish a direct causal link between anesthesia exposure and neurodevelopmental deficits in children, as well as to investigate potential mechanisms. Therefore, we designed a study in NHPs that were anesthetized during infancy under conditions that resemble those in the pediatric operating room, to investigate alterations in behaviors and accompanying long-term structural and functional changes in the primate brain. Results from these studies have been published in separate papers^18–21^. In this commentary on our latest report^21^ we focus on 1) the potential role of neuroinflammation in the behavioral outcomes associated with early-in-life anesthesia exposure and 2) the alterations in brain functional connectivity, assessed with magnetic resonance imaging (MRI), as a mechanistic link between neuroinflammation and those behavioral alterations.

## Role of neuroinflammation in AIDN

The mechanisms underlying AIDN are not fully understood, but among them anesthesia-induced neuroinflammation has been proposed as a potential contributor to AIDN. Neuroinflammation is a common hallmark of many neurodegenerative and -developmental diseases such as Alzheimer’s disease (AD), dementia, and autism spectrum disorders (ASD)^22, 23^. Here neuroinflammation plays a role in neuronal dysfunctions including cognitive impairments in neurodegenerative diseases and behavioral alterations in ASD. The neuroinflammatory response involves activation of microglia and astrocytes and commonly elevated cytokines in the brain include interleukin-6 (IL-6) and tumor necrosis factor alpha (TNFα), among others. Several reports documented acute or chronic neuroinflammation after early-in-life anesthesia exposure over the last decade, including studies in rodents and piglets showing acute neuroinflammation with increased microglia activation as well as elevated IL-6, TNFα, and IL-1β in brain tissue after single or repeated exposure to volatile anesthetics early in life^24–33^. Studies have also reported neuroinflammation present at an adult age in rats after early-in-life exposure to sevoflurane^34, 35^. In addition, maternal exposure during late pregnancy also causes neuroinflammation in the murine fetal brains and impairments in the offspring’s neurodevelopment^36, 37^. Consequently, studies applying known anti-inflammatory substances such as inhibitors of the NFκB pathway during early-in-life exposure reported reduced acute neuroinflammation and ameliorated long-term neurodevelopmental impairments^24–30, 37^. Based on the beneficial effects of pharmacologically reducing the acute inflammatory response of the brain, it’s reasonable to conclude that neuroinflammation plays a significant role in mediating the neurodevelopmental impairments of early-in-life anesthesia exposure in rodents. In NHPs, microglial activation has been detected in infants after exposure to isoflurane using positron emission tomography (PET)^38^. This inflammatory response lasted for up to 7 days, suggesting that neuroinflammation in the primate brain may play a significant role in the neurodevelopmental impairments that develop later in life. We provided first evidence that neuroinflammation is present in brains of juvenile (2-year-old) NHPs that were exposed to isoflurane early in life (on post-natal day 6). This was revealed as increased GFAP (Glial Fibrillary Acidic Protein) staining of specific brain areas, indicating increased astrocyte activation at this age^20^. This long-term astrogliosis was found in brain areas known to play an important role for the behavioral alterations the animals displayed at this juvenile age^19^, suggesting a role for chronic neuroinflammation in mediating the behavioral impairments in NHPs. However, the mechanisms linking neuroinflammation with neurodevelopmental impairments after early-in-life anesthesia exposure were not investigated either in NHPs or in other animals.

## Brain functional connectivity: a link between neuroinflammation and AIDN

Neuronal dysfunction can be reflected as alterations in brain functional connectivity that can be measured by resting-state functional connectivity magnetic resonance imaging (rs-fcMRI). In clinical studies with ASD, AD, and dementia patients, abnormal functional connectivity has been found to be associated with worse cognitive deficits or behavioral impairments and higher levels of neuroinflammation (microglia activation)^39–41^. This suggests that altered functional connectivity not only mediates cognitive deficits and impairments in behaviors, but that it is also linked to neuroinflammation. In ASD patients, extended microglial activation was suggested to be linked with a loss of connections between different brain areas or to result in functional underconnectivity^42^. Astrocytes also have been implicated in supporting functional connectivity in the healthy brain while driving abnormal connectivities in neuropsychiatric diseases with chronic neuronal dysfunctions^43, 44^. To investigate a possible mechanism linking our findings of behavioral alterations and neuroinflammation in the juvenile NHPs that were exposed to anesthesia during infancy, we followed a seed-based approach of rs-fcMRI analysis and found altered brain functional connectivity between the amygdala with other brain areas [the primary auditory cortex, the premotor cortex, and the posterior cingulate cortex (PCC)]^21^. We chose the amygdala as a seed area because it showed robust astrogliosis in the NHPs at this age^20^. In addition, it is an important hub for the processing of social behaviors which appeared impaired after early-in-life anesthesia exposure as evidenced by the decreased close-social behavior of the NHPs at the same age^19^. This increased functional connectivity of the amygdala was positively correlated with the increased astrogliosis, suggesting that chronic activation of astrocytes may have contributed to the alterations in functional connectivity of the amygdala and to the behavioral impairments in the juveniles exposed to anesthesia during infancy. Thus, our neuroimaging study in the same cohort of juvenile NHPs provides novel insights to better understand the underlying mechanisms of AIDN by linking neuroinflammation with alterations in brain functional connectivity and behavioral impairments. While, at this point, we do not have direct evidence that the histopathological and neuroimaging changes in NHPs after early-in-life anesthesia exposure lead to the behavioral impairments, studies in rodents provide some evidence that altered astrocytes activity has a causal role in modulating behaviors (recently reviewed in^45–47^). Depending on the specific brain area, alterations in astrocyte activity have been reported to modulate specific behaviors. For example, optogenetic activation of hippocampal astrocytes altered anxious behavior in mice exposed to an anxiogenic environment^48^. Another rodent study showed that selective activation of astrocytes in the striatum resulted in behavioral changes that resemble the symptoms of attention-deficit hyperactivity disorder (ADHD)^49^. In a rodent model of depression, inhibition of astrocyte activation ameliorated the depression-like behaviors^50^. In addition, a recent study using an induced pluripotent stem cell (iPSC) approach demonstrated that ASD patient-derived astrocytes when transplanted into the healthy mouse brain induce some of the behavioral impairments associated with ASD, thereby providing evidence for the astrocyte involvement in the etiology of those behaviors^51^. The amygdala also plays an important role in anxiety, which also is known to be affected by early-in-life anesthesia exposure^6, 7, 18^. Increased anxiety was found in our cohort of NHPs at the age of 1 year^18^. However, a relationship between anxiety and alterations in brain functional connectivity (and neuroinflammation) could not be established as the animals were not scanned at this age. Our rs-fcMRI analysis in the 2-year-old NHPs showed altered functional connectivity between the amygdala and the PCC, which itself is involved in social behavior. Therefore, additional analysis using the PCC as seed unsurprisingly detected altered functional connectivities with three other brain areas, and these alterations were correlated with the decreased close-social behavior. Unfortunately, the PCC was not available for our histopathological assessments, so the link between neuroinflammation and functional connectivity alterations in this area needs further investigation.

## Conclusion

While we acknowledge that our NHP cohort had a small number of animals per group and we want to be cautious about overinterpreting results, our findings suggest that chronic astroglia activation (neuroinflammation) in the amygdala (and possibly other brain areas) is associated with early-in-life anesthesia exposure and may play a role in the behavioral alterations of juvenile NHPs. In addition, alterations in brain functional connectivity of the amygdala (and possible other brain areas) provide a mechanistic link between neuroinflammation and behavioral alterations. Further studies with larger numbers of NHPs are encouraged to confirm these findings, as well as new studies using neuroinflammation modulating interventions (e.g., using anti-inflammatory drugs) to establish the causality between the neuroinflammation and the behavioral alterations associated with early-in-life anesthesia exposure.

## References

[1] Jevtovic-Todorovic V, Hartman RE, Izumi Y, Early exposure to common anesthetic agents causes widespread neurodegeneration in the developing rat brain and persistent learning deficits. J Neurosci 2003; 23: 876–82. 12574416 10.1523/JNEUROSCI.23-03-00876.2003 PMC6741934

[2] Brambrink AM, Back SA, Riddle A, Isoflurane-induced apoptosis of oligodendrocytes in the neonatal primate brain. Ann Neurol 2012; 72: 525–35. 23109147 10.1002/ana.23652 PMC3490441

[3] Brambrink AM, Evers AS, Avidan MS, Isoflurane-induced neuroapoptosis in the neonatal rhesus macaque brain. Anesthesiology 2010; 112: 834–41. 20234312 10.1097/ALN.0b013e3181d049cd PMC3962067

[4] Creeley C, Dikranian K, Dissen G, Propofol-induced apoptosis of neurones and oligodendrocytes in fetal and neonatal rhesus macaque brain. Br J Anaesth 2013; 110 Suppl 1: i29–38. 23722059 10.1093/bja/aet173 PMC3667347

[5] Paule MG, Li M, Allen RR, Ketamine anesthesia during the first week of life can cause long-lasting cognitive deficits in rhesus monkeys. Neurotoxicol Teratol 2011; 33: 220–30. 21241795 10.1016/j.ntt.2011.01.001 PMC3071878

[6] Raper J, Alvarado MC, Murphy KL, Multiple Anesthetic Exposure in Infant Monkeys Alters Emotional Reactivity to an Acute Stressor. Anesthesiology 2015; 123: 1084–92. 26313293 10.1097/ALN.0000000000000851 PMC4618245

[7] Raper J, De Biasio JC, Murphy KL, Persistent alteration in behavioural reactivity to a mild social stressor in rhesus monkeys repeatedly exposed to sevoflurane in infancy. Br J Anaesth 2018; 120: 761–7. 29576116 10.1016/j.bja.2018.01.014 PMC6200105

[8] Talpos JC, Chelonis JJ, Li M, Early life exposure to extended general anesthesia with isoflurane and nitrous oxide reduces responsivity on a cognitive test battery in the nonhuman primate. Neurotoxicology 2019; 70: 80–90. 30445043 10.1016/j.neuro.2018.11.005

[9] DiMaggio C, Sun LS, Li G. Early childhood exposure to anesthesia and risk of developmental and behavioral disorders in a sibling birth cohort. Anesth Analg 2011; 113: 1143–51. 21415431 10.1213/ANE.0b013e3182147f42 PMC3164160

[10] Flick RP, Katusic SK, Colligan RC, Cognitive and behavioral outcomes after early exposure to anesthesia and surgery. Pediatrics 2011; 128: e1053–61. 21969289 10.1542/peds.2011-0351 PMC3307194

[11] Glatz P, Sandin RH, Pedersen NL, Association of Anesthesia and Surgery During Childhood With Long-term Academic Performance. JAMA Pediatr 2017; 171: e163470. 27820621 10.1001/jamapediatrics.2016.3470

[12] Ing C, DiMaggio C, Whitehouse A, Long-term differences in language and cognitive function after childhood exposure to anesthesia. Pediatrics 2012; 130: e476–85. 22908104 10.1542/peds.2011-3822

[13] Wilder RT, Flick RP, Sprung J, Early exposure to anesthesia and learning disabilities in a population-based birth cohort. Anesthesiology 2009; 110: 796–804. 19293700 10.1097/01.anes.0000344728.34332.5d PMC2729550

[14] McCann ME, de Graaff JC, Dorris L, Neurodevelopmental outcome at 5 years of age after general anaesthesia or awake-regional anaesthesia in infancy (GAS): an international, multicentre, randomised, controlled equivalence trial. Lancet 2019; 393: 664–77. 30782342 10.1016/S0140-6736(18)32485-1 PMC6500739

[15] Sun LS, Li G, Miller TL, Association Between a Single General Anesthesia Exposure Before Age 36 Months and Neurocognitive Outcomes in Later Childhood. JAMA 2016; 315: 2312–20 27272582 10.1001/jama.2016.6967 PMC5316422

[16] Warner DO, Zaccariello MJ, Katusic SK, Neuropsychological and Behavioral Outcomes after Exposure of Young Children to Procedures Requiring General Anesthesia: The Mayo Anesthesia Safety in Kids (MASK) Study. Anesthesiology 2018; 129: 89–105. 29672337 10.1097/ALN.0000000000002232 PMC6008202

[17] Ing C, Jackson WM, Zaccariello MJ, Prospectively assessed neurodevelopmental outcomes in studies of anaesthetic neurotoxicity in children: a systematic review and meta-analysis. Br J Anaesth 2021; 126: 433–44. 33250180 10.1016/j.bja.2020.10.022 PMC8040118

[18] Coleman K, Robertson ND, Dissen GA, Isoflurane Anesthesia Has Long-term Consequences on Motor and Behavioral Development in Infant Rhesus Macaques. Anesthesiology 2017; 126: 74–84. 27749311 10.1097/ALN.0000000000001383 PMC5159295

[19] Neudecker V, Perez-Zoghbi JF, Coleman K, Infant isoflurane exposure affects social behaviours, but does not impair specific cognitive domains in juvenile non-human primates. Br J Anaesth 2020. 10.1016/j.bja.2020.10.015 PMC8040121 33198945

[20] Neudecker V, Perez-Zoghbi JF, Martin LD, Astrogliosis in juvenile non-human primates 2 years after infant anaesthesia exposure. Br J Anaesth 2021; 127: 447–57. 34266661 10.1016/j.bja.2021.04.034 PMC8451238

[21] Neudecker V, Perez-Zoghbi JF, Miranda-Domínguez O, Early-in-life isoflurane exposure alters resting-state functional connectivity in juvenile non-human primates. Br J Anaesth 2023. 10.1016/j.bja.2023.07.031 PMC10687619 37714750

[22] Lyman M, Lloyd DG, Ji X, Neuroinflammation: the role and consequences. Neurosci Res 2014; 79: 1–12. 24144733 10.1016/j.neures.2013.10.004

[23] Xiong Y, Chen J, Li Y. Microglia and astrocytes underlie neuroinflammation and synaptic susceptibility in autism spectrum disorder. Front Neurosci 2023; 17: 1125428. 37021129 10.3389/fnins.2023.1125428 PMC10067592

[24] Dai J, Li X, Wang C, Repeated neonatal sevoflurane induced neurocognitive impairment through NF-kappaB-mediated pyroptosis. J Neuroinflammation 2021; 18: 180. 34419096 10.1186/s12974-021-02233-9 PMC8380327

[25] Ji MH, Qiu LL, Yang JJ, Pre-administration of curcumin prevents neonatal sevoflurane exposure-induced neurobehavioral abnormalities in mice. Neurotoxicology 2015; 46: 155–64. 25447320 10.1016/j.neuro.2014.11.003

[26] Pang X, Zhang P, Zhou Y, Dexmedetomidine pretreatment attenuates isoflurane-induced neurotoxicity via inhibiting the TLR2/NF-kappaB signaling pathway in neonatal rats. Exp Mol Pathol 2020; 112: 104328. 31678237 10.1016/j.yexmp.2019.104328

[27] Shen X, Dong Y, Xu Z, Selective anesthesia-induced neuroinflammation in developing mouse brain and cognitive impairment. Anesthesiology 2013; 118: 502–15. 23314110 10.1097/ALN.0b013e3182834d77 PMC3580002

[28] Shi Y, Wang G, Li J, Hydrogen gas attenuates sevoflurane neurotoxicity through inhibiting nuclear factor kappa-light-chain-enhancer of activated B cells signaling and proinflammatory cytokine release in neonatal rats. Neuroreport 2017; 28: 1170–5. 28926473 10.1097/WNR.0000000000000899

[29] Wang Y, Wang C, Zhang Y, Pre-administration of luteoline attenuates neonatal sevoflurane-induced neurotoxicity in mice. Acta Histochem 2019; 121: 500–7. 31006528 10.1016/j.acthis.2019.04.004

[30] Wu N, Liu H, Lv X, Neobaicalein prevents isoflurane anesthesia-induced cognitive impairment in neonatal mice via regulating CREB1. Clinics (Sao Paulo) 2023; 78: 100201. 37120983 10.1016/j.clinsp.2023.100201 PMC10173397

[31] Broad KD, Hassell J, Fleiss B, Isoflurane Exposure Induces Cell Death, Microglial Activation and Modifies the Expression of Genes Supporting Neurodevelopment and Cognitive Function in the Male Newborn Piglet Brain. PLoS One 2016; 11: e0166784. 27898690 10.1371/journal.pone.0166784 PMC5127656

[32] Tao G, Zhang J, Zhang L, Sevoflurane induces tau phosphorylation and glycogen synthase kinase 3beta activation in young mice. Anesthesiology 2014; 121: 510–27. 24787352 10.1097/ALN.0000000000000278 PMC4165789

[33] Zhang J, Dong Y, Lining H, Interaction of Tau, IL-6 and mitochondria on synapse and cognition following sevoflurane anesthesia in young mice. Brain Behav Immun Health 2020; 8: 100133. 34589883 10.1016/j.bbih.2020.100133 PMC8474534

[34] Hogarth K, Vanama RB, Stratmann G, Singular and short-term anesthesia exposure in the developing brain induces persistent neuronal changes consistent with chronic neurodegenerative disease. Sci Rep 2021; 11: 5673. 33707598 10.1038/s41598-021-85125-5 PMC7952562

[35] Wang F, Li C, Shao J, Sevoflurane induces inflammation of microglia in hippocampus of neonatal rats by inhibiting Wnt/beta-Catenin/CaMKIV pathway. J Pharmacol Sci 2021; 146: 105–15. 33941321 10.1016/j.jphs.2021.02.004

[36] Hirotsu A, Iwata Y, Tatsumi K, Maternal exposure to volatile anesthetics induces IL-6 in fetal brains and affects neuronal development. Eur J Pharmacol 2019; 863: 172682. 31545984 10.1016/j.ejphar.2019.172682

[37] Zheng H, Dong Y, Xu Z, Sevoflurane anesthesia in pregnant mice induces neurotoxicity in fetal and offspring mice. Anesthesiology 2013; 118: 516–26. 23314109 10.1097/ALN.0b013e3182834d5d PMC3580035

[38] Zhang X, Liu S, Newport GD, In Vivo Monitoring of Sevoflurane-induced Adverse Effects in Neonatal Nonhuman Primates Using Small-animal Positron Emission Tomography. Anesthesiology 2016; 125: 133–46. 27183169 10.1097/ALN.0000000000001154

[39] Nagai Y, Kirino E, Tanaka S, Functional connectivity in autism spectrum disorder evaluated using rs-fMRI and DKI. Cereb Cortex 2023. 10.1093/cercor/bhad451 PMC11065111 38012112

[40] Passamonti L, Tsvetanov KA, Jones PS, Neuroinflammation and Functional Connectivity in Alzheimer’s Disease: Interactive Influences on Cognitive Performance. J Neurosci 2019; 39: 7218–26. 31320450 10.1523/JNEUROSCI.2574-18.2019 PMC6733539

[41] Rauchmann BS, Brendel M, Franzmeier N, Microglial Activation and Connectivity in Alzheimer Disease and Aging. Ann Neurol 2022; 92: 768–81. 36053756 10.1002/ana.26465

[42] Rodriguez JI, Kern JK. Evidence of microglial activation in autism and its possible role in brain underconnectivity. Neuron Glia Biol 2011; 7: 205–13. 22874006 10.1017/S1740925X12000142 PMC3523548

[43] Liu J, Mo JW, Wang X, Astrocyte dysfunction drives abnormal resting-state functional connectivity in depression. Sci Adv 2022; 8: eabo2098. 36383661 10.1126/sciadv.abo2098 PMC9668300

[44] Shah D, Gsell W, Wahis J, Astrocyte calcium dysfunction causes early network hyperactivity in Alzheimer’s disease. Cell Rep 2022; 40: 111280. 36001964 10.1016/j.celrep.2022.111280 PMC9433881

[45] Brazhe A, Verisokin A, Verveyko D, Astrocytes: new evidence, new models, new roles. Biophys Rev 2023; 15: 1303–33 37975000 10.1007/s12551-023-01145-7 PMC10643736

[46] Lyon KA, Allen NJ. From Synapses to Circuits, Astrocytes Regulate Behavior. Front Neural Circuits 2021; 15: 786293 35069124 10.3389/fncir.2021.786293 PMC8772456

[47] Oliveira JF, Sardinha VM, Guerra-Gomes S, Do stars govern our actions? Astrocyte involvement in rodent behavior. Trends Neurosci 2015; 38: 535–49 26316036 10.1016/j.tins.2015.07.006

[48] Cho WH, Noh K, Lee BH, Hippocampal astrocytes modulate anxiety-like behavior. Nat Commun 2022; 13: 6536 36344520 10.1038/s41467-022-34201-z PMC9640657

[49] Nagai J, Rajbhandari AK, Gangwani MR, Hyperactivity with Disrupted Attention by Activation of an Astrocyte Synaptogenic Cue. Cell 2019; 177: 1280–92 e20 31031006 10.1016/j.cell.2019.03.019 PMC6526045

[50] Wang Y, Ni J, Zhai L, Inhibition of activated astrocyte ameliorates lipopolysaccharide- induced depressive-like behaviors. J Affect Disord 2019; 242: 52–9 30172225 10.1016/j.jad.2018.08.015

[51] Allen M, Huang BS, Notaras MJ, Astrocytes derived from ASD individuals alter behavior and destabilize neuronal activity through aberrant Ca(2+) signaling. Mol Psychiatry 2022; 27: 2470–84 35365802 10.1038/s41380-022-01486-x PMC9135629

